# Abundance of the iron containing biomolecule, heme *b*, during the progression of a spring phytoplankton bloom in a mesocosm experiment

**DOI:** 10.1371/journal.pone.0176268

**Published:** 2017-04-20

**Authors:** Jessica Bellworthy, Martha Gledhill, Mario Esposito, Eric P. Achterberg

**Affiliations:** 1 Ocean and Earth Sciences, University of Southampton, Southampton, United Kingdom; 2 Geomar Helmholtz Institute for Ocean Research, Kiel, Germany; National Cheng Kung University, TAIWAN

## Abstract

Concentrations of heme *b* were determined in a mesocosm experiment situated in Gullmar Fjord off Sweden. The mesocosm experiment lasted for ca. one hundred days and was characterised by the growth of a primary nutrient replete and a secondary nutrient deplete phytoplankton bloom. Heme *b* varied between 40 ± 10 pmol L^-1^ in the prebloom period up to a maximum of 700 ± 400 pmol L^-1^ just prior to the time of the primary chlorophyll *a* maximum. Thereafter, heme *b* concentrations decreased again to an average of 120 ± 60 pmol L^-1^. When normalised to total particulate carbon, heme *b* was most abundant during the initiation of the nutrient replete spring bloom, when ratios reached 52 ± 24 μmol mol^-1^; ten times higher than values observed both pre and post the primary bloom. Concentrations of heme *b* correlated with those of chlorophyll *a*. Nevertheless, differences were observed in the relative concentrations of the two parameters, with heme *b* concentrations increasing relative to chlorophyll *a* during the growth of the primary bloom, decreasing over the period of the secondary bloom and increasing again through the latter period of the experiment. Heme *b* abundance was therefore influenced by nutrient concentrations and also likely by changing community composition. In half of the mesocosms, *p*CO_2_ was elevated and maintained at ca.1000 μatm, however we observed no significant differences between heme *b* in plus or ambient *p*CO_2_ mesocosms, either in absolute terms, or relative to total particulate carbon and chlorophyll *a*. The results obtained in this study contribute to our understanding of the distribution of this significant component of the biogenic iron pool, and provide an iron replete coastal water end member that aids the interpretation of the distributions of heme *b* in more iron deplete open ocean waters.

## 1. Introduction

Iron is an essential element for life due to its role as an electron donor or receptor in proteins [1]. Approximately 40% of iron proteins utilise the iron containing tetrapyrrole heme as a cofactor. Heme is produced via insertion of iron into protoporphyrin (IX) during tetrapyrrole synthesis, in a process analogous to the production of chlorophyll [2, 3]. Hemes occur in a variety of different structures and are widespread in living cells [2]. Free heme, unbound to an apoprotein, is highly toxic to cells and miss-regulation can lead to severe oxidative stress [4]. As a result, biochemical pathways associated with heme are tightly regulated by complex feedback systems [2]. The function of a hemoprotein is dependent upon iron ligation, charge state and the addition of different substituents on the tetrapyrrole ring [5]. Heme *b*, also known as iron protoporphyrin IX, is a versatile and abundant heme in marine organisms, contributing to between 10 and 20% of the cellular iron pool [6] and is vital to many metabolic processes [2]. Heme *b* is found in *b* type cytochromes, cytochrome p450, catalases, peroxidases, nitrate reductase and globins [3, 7]. Heme *b* plays a role in photosynthetic and respiratory electron transport, nitrate reduction and oxygen transport and storage [2, 3] and thus there could be a connection between heme abundance and marine carbon and nitrogen biogeochemical cycling. Heme may also constitute a significant source of iron to marine bacteria, as specific heme uptake pathways have been described in many bacterial species [8–11]. Despite the importance and ubiquity of hemes within marine plankton, few studies have investigated hemes in the marine environment. Recently, heme *b* distributions in the open sea and phytoplankton monocultures have been described, and related to patterns of nutrient distributions and the resultant differences in phytoplankton species composition [6, 12, 13]. However, these studies represented a snapshot of heme *b* distributions at one point in time, and it is not known how heme *b* varies in time over the evolution of a phytoplankton bloom of mixed species composition. Understanding the relationship between heme *b* and bloom dynamics is important, in order to confidently assign large variability in heme *b* to nutrient, and in particular, iron availability [6, 12, 13].

The response of heme *b* to emerging environmental changes is also unknown. With global industrialization, the concentration of carbon dioxide (CO_2_) in Earth’s atmosphere has risen faster than previously recorded. Fossil fuel burning, cement production and deforestation have resulted in recent atmospheric *p*CO_2_ surpassing 400 μatm [14]. As the surface of the global ocean absorbs increasing quantities of CO_2_, the seawater carbonate equilibrium shifts towards lower levels of carbonate (CO_2_^3-^) and increased bicarbonate (HCO_3_^-^) and results in a decrease in seawater pH [15]. This process, termed “ocean acidification”, also changes iron availability, as iron solubility and iron ligand chemistry change as a function of pH [16–19]. Since heme *b* synthesis is dependent on the insertion of iron, heme *b* abundance could therefore also be influenced by increased CO_2_ resulting from changes in the abundance of individual hemoproteins. Thus, while photosynthetic productivity is typically reported to increase [20], individual photosynthetic proteins have differential and inconsistent responses to increased CO_2_ [21–23].

Here, we describe the abundance of heme *b* over the course of a 100 day “Kiel Off-Shore Mesocosms for future Ocean Simulation” (KOSMOS) [24] study in Gullmar Fjord, Sweden. The experiment was a large multidisciplinary program and the full details are reported in the first paper of this collection [25]. Our aims in the KOSMOS experiment were to firstly quantify and describe heme *b* abundance in an iron replete marine community over the evolution of a phytoplankton bloom and secondly to study the response of heme *b* to increased *p*CO_2_. Relationships between heme *b*, chlorophyll *a* (chl *a*), total particulate carbon (TPC) and total particulate nitrogen (TPN) were therefore examined during the mesocosm plankton bloom exposed to low and ambient pH treatments.

## 2. Materials and methods

### 2.1. Mesocosm installation, CO_2_ manipulation and maintenance

Mesocosm deployment, manipulations, and maintenance are described in detail in this collection in [25]. Briefly, an array of 10 KOSMOS units was moored in Gullmar Fjord, Sweden, in January 2013. Each unit consisted of an 18-meter deep mesocosm bag attached to a buoyed floatation frame ending in a conical-shaped sediment trap and covered above the surface by a hood. Mesocosms were closed prior to the main experiment on 7^th^ March 2013 (Day (T) = -2) when daily CTD (conductivity, temperature, density) profiles indicated a moderate salinity (29.1) inside the mesocosms. All mesocosms were subsequently bubbled with air using a spider device [24] which resulted in a well-mixed water column and the absence of density stratification.

CTD profiles and macro nutrient analysis indicated no significant differences between the initial mesocosm conditions. Five were randomly chosen as high CO_2_ replicates whilst the remaining five were left as ambient control replicates. Over the course of four CO_2_ additions (T = -1, 0, 2 and 4) mesocosms 2, 4, 6, 7 and 8 were manipulated to ca. 1000 μatm *p*CO_2_ [25]). Further CO_2_ additions were made on days 17, 46, 48, 68 and 88 in order to maintain significantly elevated *p*CO_2_ in the manipulated mesocosms.

Field work arranged through Sven Lovén Centre for Marine Sciences, Kristineberg. Permission for the fieldwork was obtained from the The County Administrative Board of Västra Götaland, (Decision 2012-12-11, Diarienummer 258-39615-2012).

### 2.2. Sampling

Vertical profiles of temperature and salinity were measured in every mesocosm and the adjacent fjord water every second day between 14.00 and 16.00 hours using a CTD (Sun and Sea technologies). Samples for chl *a*, nutrients (nitrate+nitrite, phosphate, silicate) and TPC/N were collected from each mesocosm and the adjacent fjord water every two days, and heme *b* every four days, between 09.00–12.00 hours from 10^th^ March 2013 (T1) to 14^th^ June 2013 (T97) using an Integrated 5 L Water Sampler (IWS; Hydro-Bios, Kiel, Germany), which enabled collection of integrated water samples to 18 meters depth.

Subsamples (1 L) for heme were filtered at The Sven Lovén Centre for Marine Sciences (Kristineberg, Sweden) onto 25 mm, 0.7 μm pore size glass microfiber filters (MF300, Fisher Scientific, Leicester, UK). Filters were stored at -80°C in plastic microcentrifuge tubes (Eppendorf) before laboratory analysis at National Oceanography Centre, Southampton, UK. Samples (800 mL) for chl *a* determination by HPLC were filtered (0.7 μm glass fibre filters) prior to being frozen at -80°C before analysis. Water samples for TPC/N analysis (500 mL) were filtered onto pre-combusted glass microfiber filters before storage at -20°C. Water samples for nutrient analysis were collected directly from the water sampler into 200 ml polyethylene bottles (Nalgene).

### 2.3. Analysis of nutrients, chl *a* and total particulate carbon/nitrogen

Analysis of nutrients, chl *a* and TPC/N are described in detail in [25]. Briefly, for nutrients, samples were syringe-filtered (0.45 μm acetate cellulose filters, Whatman) prior to analysis conducted on the same day using a QuAAtro autoanalyser (SEAL Analytical, ACCE software). After the 37^th^ day, concentrations of nitrate and phosphate became too low to detect with the QuAAtro autoanalyser, and were instead determined using a nanomolar nutrient system equipped with long wave guide capillary cells [26, 27]. Detection limits for the nanomolar system were 2 nmol L^-1^ for nitrate and 1 nmol L^-1^ for phosphate.

Chl *a* was analysed by HPLC after extraction into 90% acetone as described in reference [28]. Samples for TPC/N were analysed after combustion using an elemental analyser [28]. Samples were not acidified prior to analysis so reported concentrations include a contribution from particulate inorganic carbon. However, calcification was not thought to be significant in the mesocosms [25], so particulate carbon is likely to be have been mostly organic.

### 2.4. Heme *b* determination by high performance liquid chromatography

Heme *b* was quantified following the method of Gledhill [29, 30]. Heme *b* was extracted into ammoniacal detergent (2.5% Octyl gluco pyranoside in 0.02 mol L^-1^ NH_4_OH) and analysed using an Accela High Performance Liquid Chromatography system equipped with a photo-diode-array detector (HPLC-PDA, Thermo Scientific) and an electrospray ionisation mass spectrometer (ESI-MS, LTQ-Velos, Thermo Scientific). The aqueous mobile phase consisted of 0.1% nonafluoropentanoic acid (NFPA). The organic phase was a 1:1 (v:v) mixture of isopropanol and acetonitrile with the addition of 0.1% NFPA. Heme *b* was separated from other pigments using a polystyrene divinyl benzene HPLC column (PLRP-S, Agilent Technologies). A gradient of 90% aqueous: 10% organic phase to 100% organic phase over 20 minutes followed by 10 minutes isocratic elution with 100% organic phase was used. The flow rate was set to 200 μL min^-1^ with an injection volume of 100 μL. In this study we used UV absorbance at 400 nm to quantify heme *b* [29] rather than the ESI-MS signal [30], as the ESI-MS signal appeared to be suppressed by high levels of background organic material. Nevertheless the MS allowed us to confirm that the UV response was due to heme *b* [30]. The analytical detection limit (= blank + (3 × standard deviation of the blank)) was determined daily and averaged 9±8 pmol heme *b* L^-1^ seawater.

The extraction of heme *b* with an ammoniacal detergent for HPLC is known to be incomplete [6, 12] and therefore values obtained in this study are operationally defined, not fully quantitative. However, the application of identical methods as previously used to determine chl *a*: heme *b* relationships in open ocean systems [6, 12], permits a comparison to the coastal fjord of this study.

### 2.5. Statistical analysis

Graphs and statistical analysis were completed using SigmaPlot v.12.3. Values are expressed as mean ± 1 standard deviation for the entire experimental period. As all parameters failed Shapiro Wilk normality tests (p = >0.05), the non-parametric One Way ANOVA on ranks was used to assess significant differences between *p*CO_2_ treatments.

## 3. Results

### 3.1. Temperature, salinity and nutrient concentrations in the mesocosms and Gullmar Fjord

**Fig 1:**
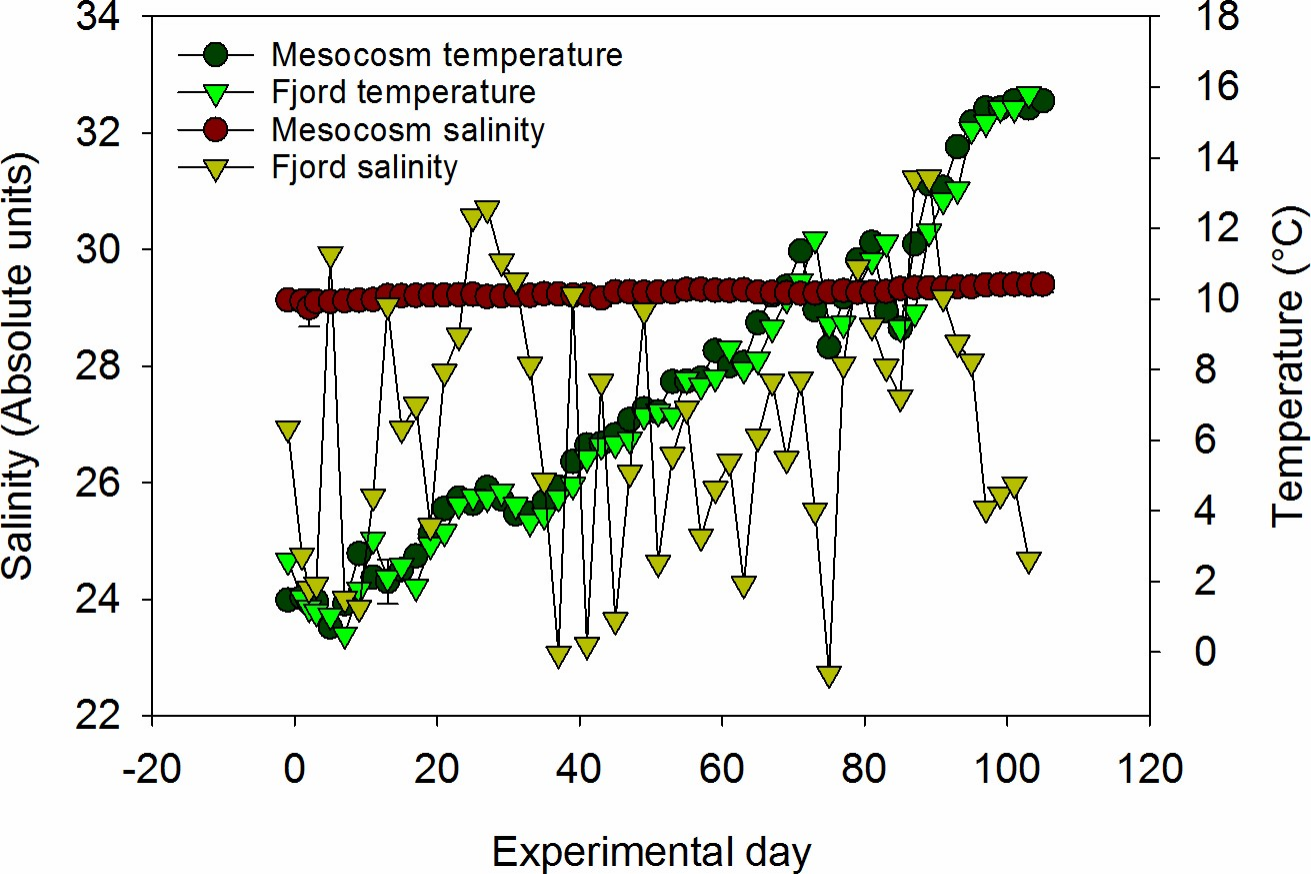
Salinity and temperature profiles in the mesocosms and fjord throughout the experiment.

**Fig 2:**
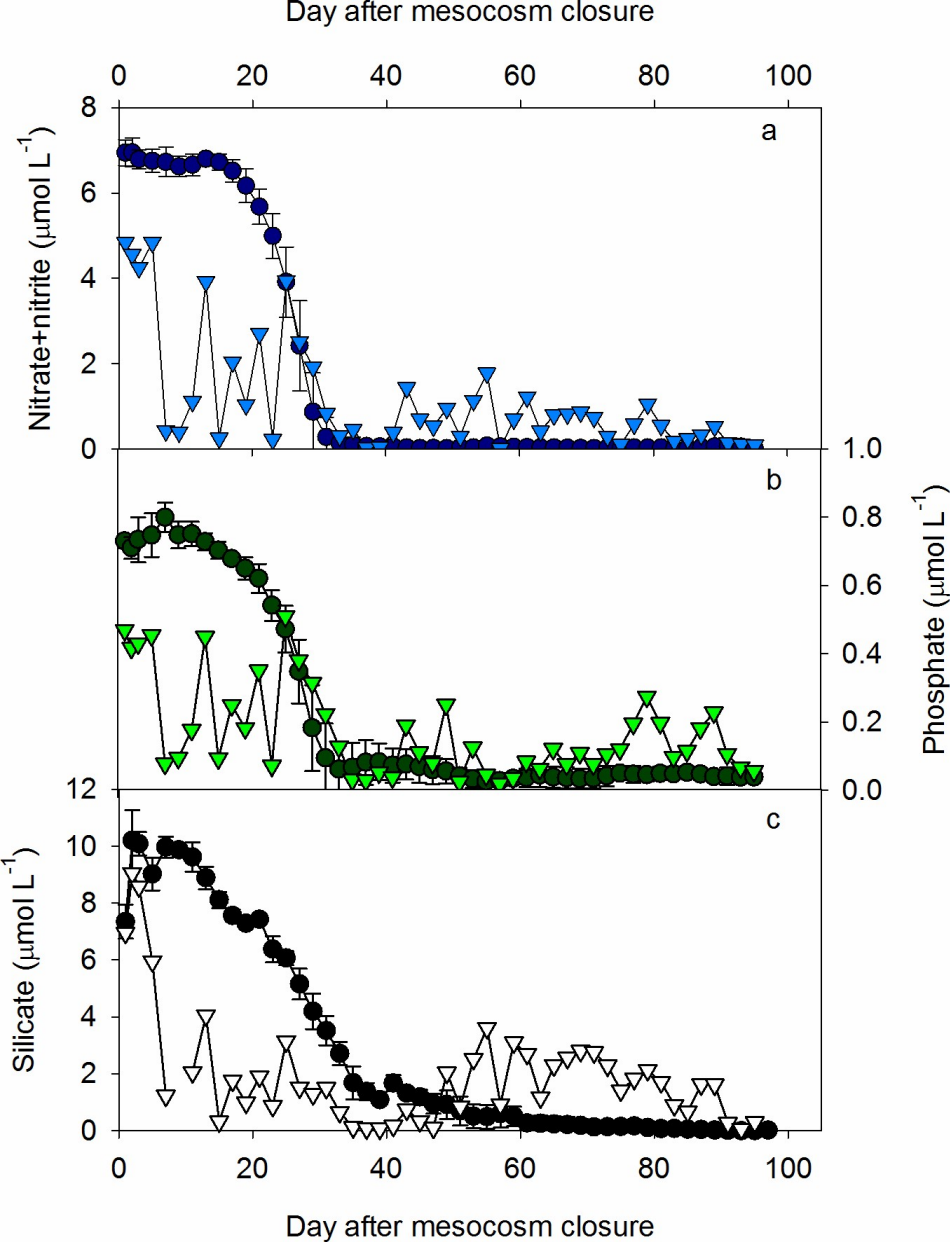
Average nutrient concentrations from a depth integrated water sampler (0–18 m). (a) nitrate + nitrite, (b) phosphate and (c) silicate in the mesocosms (circles) and adjacent fjord (triangles) throughout the experiment. Values for mesocosms are expressed as the average ± standard deviation (n = 10).

Mesocosm bags were left open for more than 48 hours after deployment to allow complete water exchange and the reduction of inter- mesocosm variability with regards to small-scale biological and chemical patchiness within the fjord. The lack of detectible differences in salinity, temperature, density, chl *a* and nutrients were a prerequisite for closing. A detailed discussion on general mesocosm conditions, including salinity, temperature and *p*CO_2_ is presented elsewhere [25]. Salinity in the mesocosms at the start of the experiment was 29.1 ± 0.1 and was stable throughout with an overall average of 29.2 ± 0.1. Temperatures increased gradually from 2.1 ± 0.15°C at the beginning to 16 ± 0°C at the end of the experiment (Fig 1). Ambient and high *p*CO2 mesocosms averaged 394.99 ± 50.89 and 789.28 ± 184.41 μatm respectively. Statistical analysis described in [25] confirmed significant differences (p = <0.001) in *p*CO_2_ between treatments, whilst replicate mesocosms within each treatment were not different (p = >0.05). However, no significant differences were observed for physical or nutrient data when *p*CO_2_ treatments were compared [25], hence in this study we present and consider only overall averages for parameters in the mesocosms. Nitrate + nitrite concentrations (hereafter termed nitrate or N for simplicity) decreased from an average of 6.9 ± 0.3 μmol L^-1^ at the beginning of the experiment (day 1) to concentrations below 0.1 μmol L^-1^ on day 35 (Fig 2A). Phosphate followed a similar trend, decreasing from 0.73 ± 0.02 μmol L^-1^ to levels below 0.1 μmol L^-1^ on day 37 (Fig 2B). Silicate concentrations also decreased over the course of the experiment from a maximum of 10.2 ± 1.0 μmol L^-1^ on day 2, although levels of silicate did not drop below 0.5 μmol L^-1^ until after day 57 (Fig 2C).

At the time of mesocosm closure, CTD profiles showed that the adjacent fjord was strongly stratified at ~ 12 meters depth. *p*CO_2_ averaged 361.8 ± 47.55 μatm in the fjord during the course of the experiment. Salinity at the fjord sampling point fluctuated between 22 and 31, reflecting the more dynamic mixing regime of the fjord proper. Temperatures, however, were very similar to those observed in the mesocosms, and increased from 1.9 to 16.1°C (Fig 1). Nutrient concentrations in the surface waters of the fjord were all slightly lower than those in the mesocosms on T = 0 (Fig 2A–2C), likely a result of changes in water mass in the fjord between the day mesocosms were closed (T = -2) and the start of the experiment. A decrease in N and P was observed between days 25 and 31 that tracked the decrease observed in the mesocosms over the same time period. After day 31 concentrations of N, P and Si fluctuated, albeit at lower concentration than observed at the beginning of the experiment. The concentration of P (but not N or Si) correlated with salinity (r = 0.61, p < 0.01, n = 34) indicating that physical mixing processes within the fjord proper were likely to be at least partially driving the observed fluctuations.

### 3.2. Average chl *a*, POC, PON, and heme *b* in the mesocosms and fjord

**Fig 3:**
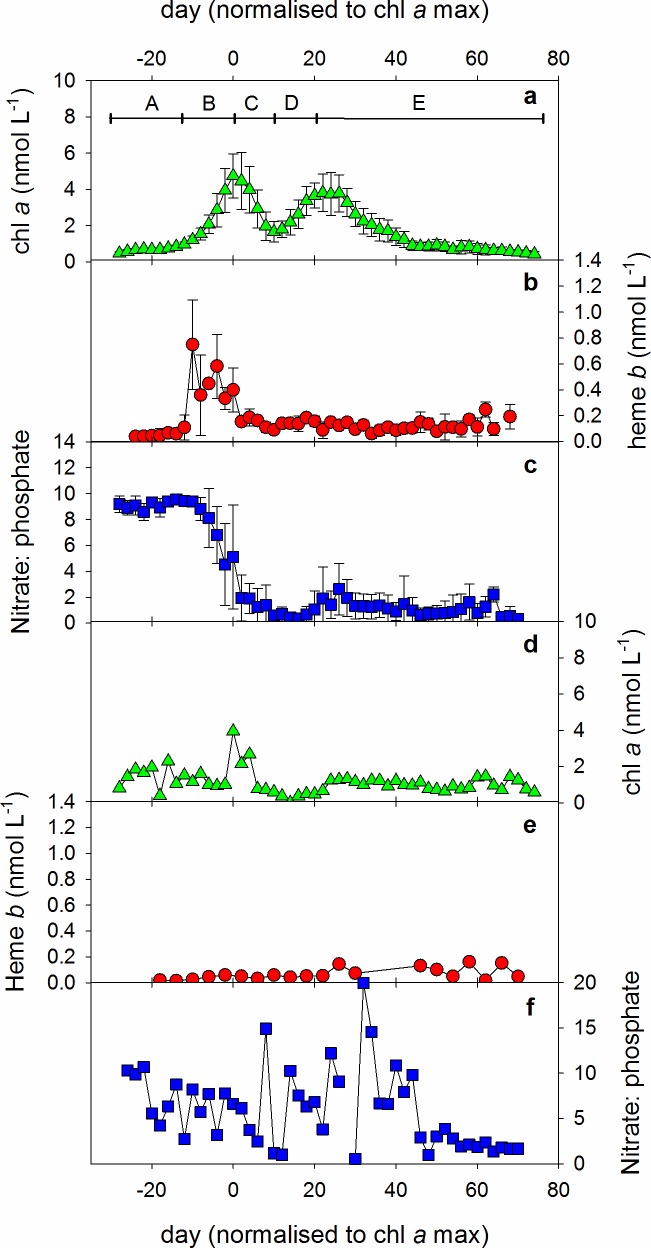
(a) Chlorophyll *a* concentrations, (b) heme *b* concentrations and (c) (nitrate + nitrite): phosphate ratio (N:P) in the mesocosms as function of time. Values are expressed as averages plus or minus the standard deviation. For chlorophyll *a* and N:P, n = 10, for heme *b*, n varied between 1 and 6, averaging 4.6, depending on how many samples were taken on any given day relative to the chlorophyll *a* maximum. (d) Chlorophyll *a* concentration, (e) heme *b* concentration and (f) N:P ratio in the Gullmar Fjord over time. Only one sample was collected from the fjord each sampling day. Day 0 was defined as the day that chlorophyll *a* reached a maximum and occurred between the 6^th^ and 10^th^ April (average 30.2 ± 1.9 days after mesocosm closure) in the mesocosms and on the 7th April 2013 in the fjord. The experiment was divided into five different periods based on the changes in chlorophyll *a* as denoted by the capital letters A—E.

Chl *a* concentrations rose from an average of 0.40 ± 0.02 nmol L^-1^ one day after mesocosm closure up to a maximum of 4.5 ± 1.4 nmol L^-1^ 33 days after mesocosm closure (S1 Table). At the start of the experiment, average chl *a* concentrations were indistinguishable between *p*CO_2_ treatments and no statistical difference was observed in the daily average chl *a* concentrations between treatments. Maximum chl *a* concentrations in the mesocosms were observed an average 30.4 ± 1.3 days after the mesocosm bags were closed, however as chl *a* peaked on slightly different days after mesocosm closure in different mesocosms, we normalised the timescale for each mesocosm to the day of maximum observed chl *a* (= day 0). Normalisation of time to the day of maximum chl *a* allowed us to examine the bloom progression consistently in the mesocosms, and to resolve any temporal differences between chl *a* and heme *b* concentrations. Adopting this approach showed that chl *a* in the mesocosms increased exponentially for approximately 14 days prior to peaking at an average value of 4.7 ± 1.2 nmol L^-1^ (Fig 3A). A rapid decrease in chl *a* concentration was recorded before a secondary maximum occurred after a further 22 days, with an average chl *a* concentration of 3.8 ± 1.0 nmol L^-1^. The experimental period was split into five stages based on the chl *a* concentrations (Fig 3A): (A) Days -30 to -14 encompassed the initial pre-primary bloom period of slight increase/stable chl *a*, (B) days -12 to 0 incorporated the growth of the primary bloom, (C) days 2–10 included the decline of the primary bloom (D) days 12–20 encompassed the growth of the second bloom and (E) days 22–76 characterized by the gradual decline of chl *a* until the end of the experiment. We use a different notation than those used elsewhere in this collection as we have normalised bloom progression to chl *a*, however our stages are broadly comparable to those described in [25]. (A) is thus similar to period I, (B) and (C) encompass period II, (D) corresponds with the first part of period III and (E), the second half of period III and period IV.

Average heme *b* concentrations for each day normalised to the time of the chlorophyll *a* maximum are presented in Fig 3B and given in S1 Table. Initial mesocosm heme *b* concentrations remained below 100 pmol L^-1^ in period (A) (Fig 3B), averaging 40 ± 10 pmol L^-1^ (n = 6). Heme *b* concentrations then increased sharply over a four day period, peaking at 700 ± 400 pmol L^-1^, 4.4 ± 4.3 days before the chl *a* maximum was observed. In period (C), heme *b* concentrations decreased until eight days after the chl *a* maximum, at which point chl *a* also reached a between bloom minimum. However, in contrast to chl *a*, heme *b* remained relatively constant in the mesocosms in periods (D) and (E) after the primary bloom period, averaging 120 ± 60 pmol L^-1^ overall (n = 133).

The decrease in chl *a* and heme *b* coincided with a decrease in the N:P ratio in the mescocosm from an initial period (A) average of 9.1 ± 0.3 (n = 9) (Fig 3C). N:P ratios were slightly lower in period (B) and began to decrease began four days before the peak in chl *a* concentrations. Period (C) was characterised by a marked decrease in N:P ratios until nutrient concentrations fell below 0.1 μmol L^-1^ in period (D). The decrease in N:P suggests that nitrate + nitrite was consumed more rapidly than phosphate and that the bloom in the mesocosm was thus nitrate limited.

In period (A), both TPC and TPN were relatively stable, averaging 15 ± 1 and 2.0 ± 0.2 μmol L^-1^ respectively (n = 79). Total particulate carbon and nitrogen then increased and reached maxima of 50 ± 13 (n = 10) and 6.7 ± 0.7 μmol L^-1^ (n = 10) in period (B), 4 days after the chl *a* maximum. After reaching a maximum, TPC and TPN both declined gradually with time, before increasing again to a secondary maximum coincident with the second chl *a* maximum on day 22 (S1 Table).

In the adjacent fjord, the maximum chl *a* concentration was observed 29 days after the mesocosm bags were closed and thus coincided temporally with the maximum chl *a* concentration in the mesocosms (S2 Table). However, in contrast to the mesocosms, during the early phase of the experimental period, chl *a* in the fjord fluctuated between 0.4 and 2.3 nmol L^-1^, likely a result of the more variable mixing regime observed in the fjord itself (Fig 3D).

Heme *b* concentrations in the fjord at the start of the experiment were similar in magnitude to those within the mesocosms. During the course of the experiment, heme *b* concentrations ranged from below the detection limit up to a maximum fjord heme *b* concentration of 160 pmol L^-1^, occurring in the final days of the experiment (Fig 3E, S2 Table). Heme *b* concentrations did not reach a distinct maximum in the fjord, despite increased chl *a* concentrations (Fig 3E). The ratio of N: P in the samples collected from the Gullmar Fjord was greater than 10 for the first five days of the experiment (Fig 3F). Thereafter, N:P ratios were generally less than 10 until higher and variable values were again observed 10–24 days after the chl *a* maximum.

TPC and TPN concentrations in the fjord ranged between 13.1 and 58.3 μmol L^-1^, and 1.9 and 5.7 μmol L^-1^ respectively (S2 Table). Maximum TPC and TPN concentrations were observed 7 days after the mesocosms were closed and thus occurred earlier in the fjord compared to the mesocosms. This maximum was characterised by high C:N ratio (ca. 11) and coincided with low salinity and temperature, suggesting that this maximum was again associated with dynamic water mass movements within the fjord, rather than directly related to bloom dynamics.

### 3.3. Relative changes in TPC, chl *a* and heme *b*

**Fig 4:**
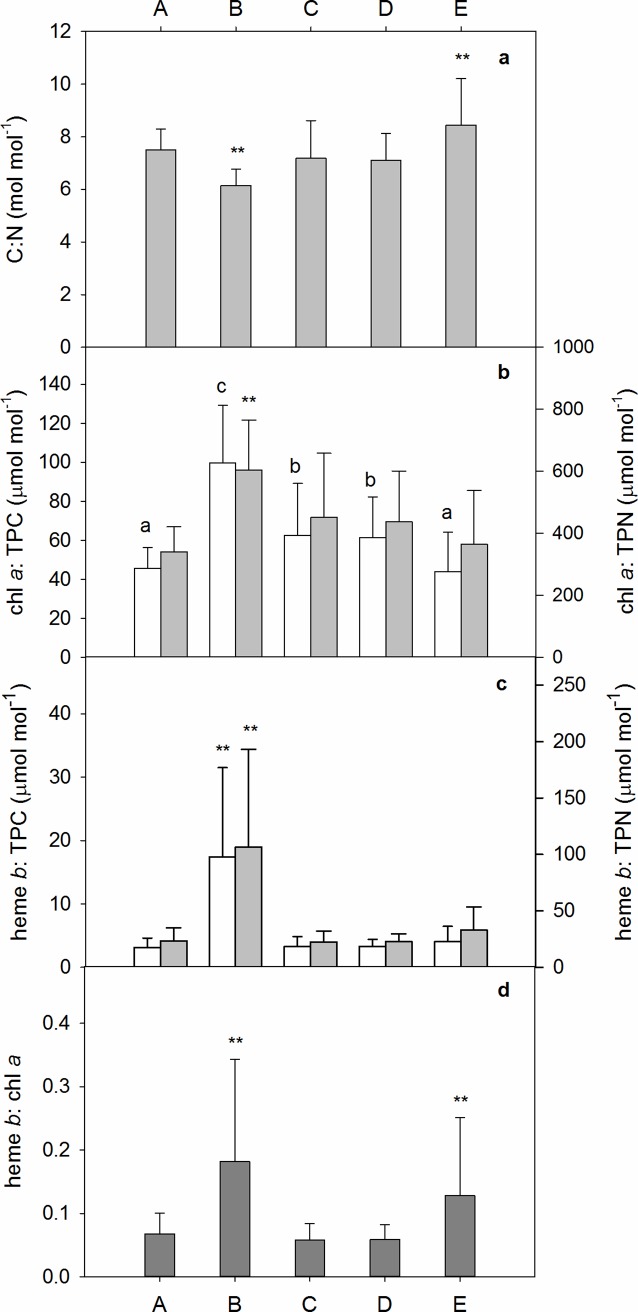
**Mean (± S.D.) ratios of key variables during each defined period in the mesocosms (A—E).** Asterisks indicate periods that were significantly different. a: C:N, b: chl a: TPC (white bars) and chl a: TPN (grey bars), bars with the same letter are not significantly different from one another, c: heme b: TPC (white bars) and heme b: TPN (grey bars), d: heme b: chl a

In the mesocosms, there was a strong correlation between TPC and TPN, (Table 1) and the C:N ratio averaged 7.7 ± 1.6 overall (n = 480), supporting the suggestion that inorganic carbon made only a minor contribution to the TPC pool. Nevertheless C:N ratios were significantly lower (6.1±0.6, p<0.01, n = 50) in period (B) compared to all other periods and significantly higher in period (E) (8.4±1.8, p<0.01, n = 225, Fig 4A). Chl *a* concentrations correlated with both TPC and TPN (Table 1), although there was some variability in the chl *a*: TPC ratios with significantly lower averages observed for periods (A) and (E), and significantly higher values observed in period (B) (Fig 4B). Correlation between chl *a* and TPN was stronger than for chl *a* and TPC (Table 1), and was reflected in the results for the ANOVA on the different bloom periods as chl *a*: TPN ratios were only significantly higher in period (B) (Fig 4B). Heme *b* concentrations also correlated with TPC, TPN and chl *a*, however, the correlation was weaker because heme *b*: TPC, TPN and chl *a* ratios increased by approximately four fold in the primary bloom period (period (B), Fig 4B and 4C). Average heme *b* concentrations in the mesocosms were never more than 100 times lower than the average chl *a* concentrations; heme *b*: chl *a* was thus higher than observed in previous studies [6, 12]. After the first bloom period, heme *b*: chl *a* ratios dropped in periods (C) and (D) but rose again in period (E) to values approaching those observed at the peak of the primary bloom (Fig 4D).

**Table 1:** Correlation coefficients observed for total particulate carbon (TPC), total particulate nitrogen (TPN), chlorophyll *a* and heme *b* concentrations in the mesocosms and Gullmar Fjord over the entire course of the experiment.

		TPN	Chlorophyll *a*	Heme *b*
Mesocosms	TPC	0.91, p<0.01, n = 481	0.75, p<0.01 n = 481	0.29, p<0.01, n = 215
	TPN		0.82, p<0.01 n = 481	0.39, p<0.01, n = 215
	Chlorophyll *a*			0.46, p<0.01, n = 216
Fjord	TPC	0.81, p<0.01, n = 50	0.33, p = 0.02 n = 50	-0.2, p>0.5, n = 20
	TPN		0.29, p = 0.04 n = 50	-0.05, p>0.5, n = 20
	Chlorophyll *a*			-0.03, p>0.5, n = 20

**Fig 5:**
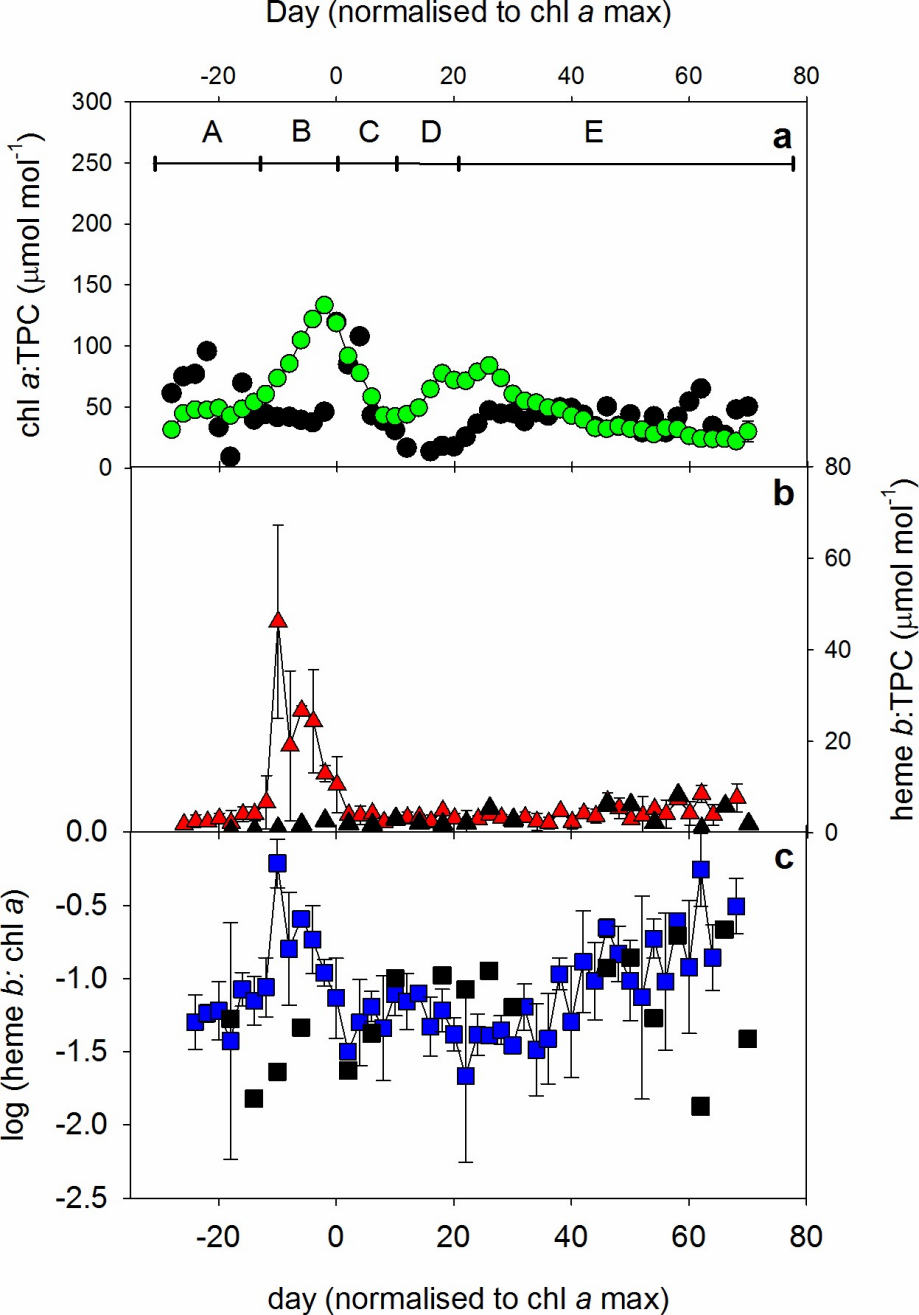
(a) Chlorophyll *a*: TPC, (b) heme *b*: TPC and (c) log(heme *b*: chl *a*) ratios observed in the mesocosms (coloured symbols) and fjord (black symbols) over time normalized to the day of maximum chlorophyll *a*. Day 0 was defined as the day that chlorophyll *a* reached a maximum and occurred between the 6^th^ and 10^th^ April in the mesocosms (an average 30.2 ± 1.9 days after mesocosm closure) and on the 7th April 2013 in the fjord. Values are expressed as the average ± standard deviation. The number of samples, n, depended on the number of samples occurring on any given day relative to the chlorophyll *a* maximum and was between 2–4 for chlorophyll *a* and heme *b*: TPC, and 2–6 for chlorophyll *a*: heme *b*. One sample was collected from the fjord each sampling day. The experiment was divided into five different periods based on the changes in chlorophyll *a* as denoted by the capital letters A—E.

In the fjord, TPN again correlated with TPC so that the ratio of C:N averaged 7.1 ± 1.2 overall (n = 50) and was thus slightly lower than the average observed in the mesocosms. In contrast to the mesocosms, neither chl *a* nor heme *b* correlated with TPC, TPN or each other in the fjord at the 99% confidence level (Table 1). This is likely influenced by the lower number of samples collected in the fjord (Table 1). Nevertheless, the range in chl *a*: TPC ratios were similar in the fjord to those observed in the mesocosms and highest chl *a*: TPC was also recorded in the fjord in the period when chl *a* was higher (Fig 5A). Heme *b*: TPC ratios observed in the fjord were, however, lower than those observed in the mesocosms (Fig 5B). Even so, heme *b*: TPC ratios were higher than those reported previously for open ocean regions [6, 12]. The relative lower abundance of heme *b* in the fjord was confirmed by comparison with chl *a*, as chl *a* concentrations were observed to be as much as 260 times higher than heme *b* in the period where chl *a* concentrations were maximal (Fig 5C).

## 4. Discussion

### 4.1. Heme *b* during the evolution of a phytoplankton bloom of mixed community composition

In this study, we investigated for the first time the temporal variability of heme *b* over the course of a phytoplankton bloom with a mixed community composition occurring during a mesocosm experiment. As a component of both the respiratory and photosynthetic electron transport chains, heme *b* occurs in all planktonic marine organisms. We filtered our samples through a 0.μm filter, and so theoretically exclude smaller microbes (which are likely to be mostly heterotrophs) from our samples. Nevertheless, the evolution of a bloom through primary to regenerative production, and through fluctuations in phototrophic and heterotrophic communities, typically observed in mesocosm experiments [31–33], might be expected to impact on heme *b* concentrations, and the relationship between heme *b* and other bulk components of the biogenic carbon pool (POC, PON, chl *a*). Fluctuations in heme *b* could be influenced by both community composition [13] and by changes in ambient nutrient concentrations [6, 34]. In general, the mesocosm community composition was dominated by small (2–5 μm) and large diatoms (>200 μm), and chlorophytes, with a change in species composition observed between the first and second bloom period [25]. The first bloom terminated as result of the reduction in nitrate concentrations, and community productivity thereafter was dominated by regeneration [25]. The switch from primary to regenerative production is characterised by a shift in nitrogen metabolism from the use of oxidised nitrogen sources to the use of reduced nitrogen. Such a switch also has potential impacts on heme *b* as eukaryotic nitrate reductase incorporates a *b* type cytochrome [35].

The maximum heme *b* concentration observed in the mesocosms was 44 times higher than the maximum heme *b* concentration reported previously in open waters(21 pmol L-1; [12]). This was partly a reflection of higher biomass observed in the mesocosms than typically observed in shelf or open ocean regions. For example, TPC in the mesocosms ranged between 9.6–77.3 μmol L^-1^, while reported values for the mixed layer depth in the Celtic Sea and tropical North Atlantic were 10 ± 1.6 μmol L^-1^ and ca. 2–3 μmol L^-1^ respectively [6, 12]. Heme *b* concentrations, however, were disproportionately higher relative to TPC and chl *a* and this resulted in higher heme *b*: TPC and heme *b*: chl *a* ratios being observed in this study than have previously been observed in field studies [12, 34]. Maximum average heme *b*: chl *a* and heme *b*: TPC ratios were observed several days before the chl *a* maximum at a time corresponding to the initiation of the primary phytoplankton bloom. The heme *b*: chl *a* and heme *b*: TPC ratio was also considerably higher than that observed in phytoplankton monocultures, which have previously been reported only for the end of the exponential phase [6, 13]. As the period of maximum heme *b*: chl *a* and heme *b*: TPC ratios was pre-bloom, it is likely that the relative increase in heme *b* is associated with elevated concentrations of heme *b* within the photosynthetic phytoplankton population, rather than relative changes in the abundance of heterotrophs and phototrophs or shifts in community composition. The results obtained in this study of mixed phytoplankton assemblages therefore suggest that at the time of bloom initiation, which corresponds to optimum nutrient concentrations, hemoprotein, abundance was higher in order to facilitate growth. A potential cause for the increased hemoprotein content is likely to have been the requirement for assimilatory nitrate reductase. The transient nature of the increase suggests that even before the primary bloom terminated as a result of reduced nitrate availability, the phytoplankton population switched to ammonia utilisation as a less energy demanding nutrient source. Rapid utilisation of ammonia would also be consistent with the absence of ammonia accumulation during the course of the mesocosm experiment [25]. In previous laboratory cultures, heme *b* has been shown to make up between 6 and 40% of the total cellular iron at the end of the exponential growth phase [6]. The high heme *b*: TPC ratio observed at the start of the bloom suggests that the proportion of cellular iron allocated to heme *b* and/or the cellular demand for iron could also change over the course of a phytoplankton bloom.

It was notable that heme *b*: TPC ratios remained relatively stable for the remainder of the experimental period and thus did not increase at any time during the secondary bloom, in contrast to chl *a*: TPC which increased during both bloom periods. This was likely due to the switch to regenerative production, coupled to the change in community composition over the course of the experiment. The results from this mixed community mesocosm population therefore support previous laboratory single culture results and suggest the plankton population growing under low nutrient conditions allocated energy resources away from the synthesis of hemoproteins, possibly because of the reduced need for eukaryotic nitrate reductase.

The positive correlation between heme *b* and chl *a* suggests that phytoplankton abundance had a strong influence on heme *b* concentrations. However, Fig 5C suggests that the relationship between heme *b* and chl *a* was also influenced by both nutrient abundance and changing species composition. The N:P ratio suggests that both mesocosm and fjord waters were deficient in oxidised inorganic nitrogen, with respect to requirements for the production of organic matter with classical Redfieldian N:P ratios, at the start of the experiments. As with the heme *b*: TPC ratio, heme *b* abundance increased relative to chl *a* in the first part of the primary bloom, but started to fall again as nutrient depletion set in. In the latter part of the experiment however, we observed a steady increase in the overall abundance of heme *b* relative to chl *a*, and in the variability of heme *b*: chl *a* values (Fig 5C). This is possibly due to fluctuating populations of heterotrophs such as zooplankton which contain heme *b* but not chl *a* (although heme *b* levels in marine zooplankton have yet to be determined using the protocol applied in this study). In the final phase of the experiment chl *a*: TPC decreased whilst heme *b*: TPC increased supporting the idea of a shift in species composition likely from primary producers to a greater abundance of zooplankton. It appeared that the mesocosms within this study had significant zooplankton abundance as not all TPC variability was accounted for by chl *a* alone. This is in contrast to Honey et al. [6] who assigned a minimal proportion of the heme *b* pool to zooplankton.

Taken together, the results suggest that heme *b* concentration, and the abundance of heme *b* relative to other bulk parameters of the biogenic carbon pool are influenced by the bloom phase. Thus, at the initiation of a primary bloom, heme *b* concentrations increase, and reach a maximum relative both to TPC and to chl *a*. Concentrations of heme *b* in the water column will then continue to change with biomass, but the abundance of heme *b* relative to TPC and chl *a* will decrease as the community switches to regenerated production. As the community further evolves and heterotrophs make a larger contribution, concentrations of heme *b* become more variable and increase relative to TPC and chl *a*.

The overall concentration of heme *b* in the water column and the abundance of heme *b* relative to TPC and chl *a* was lower in the Gullmar Fjord relative to the mesocosms. The fluctuations observed in salinity and the difference in overall nutrient concentrations, coupled with the earlier onset of the reduction in N:P ratios are likely linked to the different water column conditions in the fjord compared to the mesocosms. Thus the lower abundance of heme *b* in the fjord, both in absolute terms and with respect to POC and chl *a* most likely arose because of water mass movements bringing water of differing salinity, N:P ratios and overall N and P composition.

### 4.2. Effect of high *p*CO_2_ conditions

In this study, we assessed the potential impact of increased *p*CO_2_ on heme *b*. We found no statistically significant impact of increased *p*CO_2_ on heme *b* concentrations in the water column, or on the abundance of heme *b* relative to other bulk parameters associated with the biogenic carbon pool. Although iron concentrations in the fjord likely vary on both temporal and spatial scales depending on the relative input from rivers and sediments [36, 37], it is very unlikely that planktonic production here is iron limited given that previous reported surface dissolved iron in Gullmar Fjord ranged from ca. 4–40 nmol L^-1^ [36, 37]. Reallocation of heme *b* resources in response to changes in *p*CO_2_ may be more pronounced in low iron or iron limited regions [22]. Previous studies in iron replete mesocosms report no significant acidification effect upon phytoplankton species composition or succession [31–33], microzooplankton grazing [38] or copepod feeding and egg production [39]. Significant pH-effects have also failed to manifest in field experiments on microzooplankton biomass in a late North Atlantic spring bloom [40] and may further explain the lack of change in heme *b* and its relation to other parameters. In relation to iron, there are suggestions that lowered pH results in both increased solubility of iron and a higher fraction of iron in the inorganic form [17, 41]. However, there are also contrasting results suggesting that iron bioavailability may decrease [18, 19] due to increased organic binding and more work is needed to ascertain the prevailing effects of increased *p*CO_2_ upon nutrient chemistry [20]. The results obtained in this study, do not therefore rule out an impact of changes in *p*CO_2_ on open ocean, low iron or iron limited communities.

### 4.3. Comparison with previously reported heme *b* concentrations

**Fig 6:**
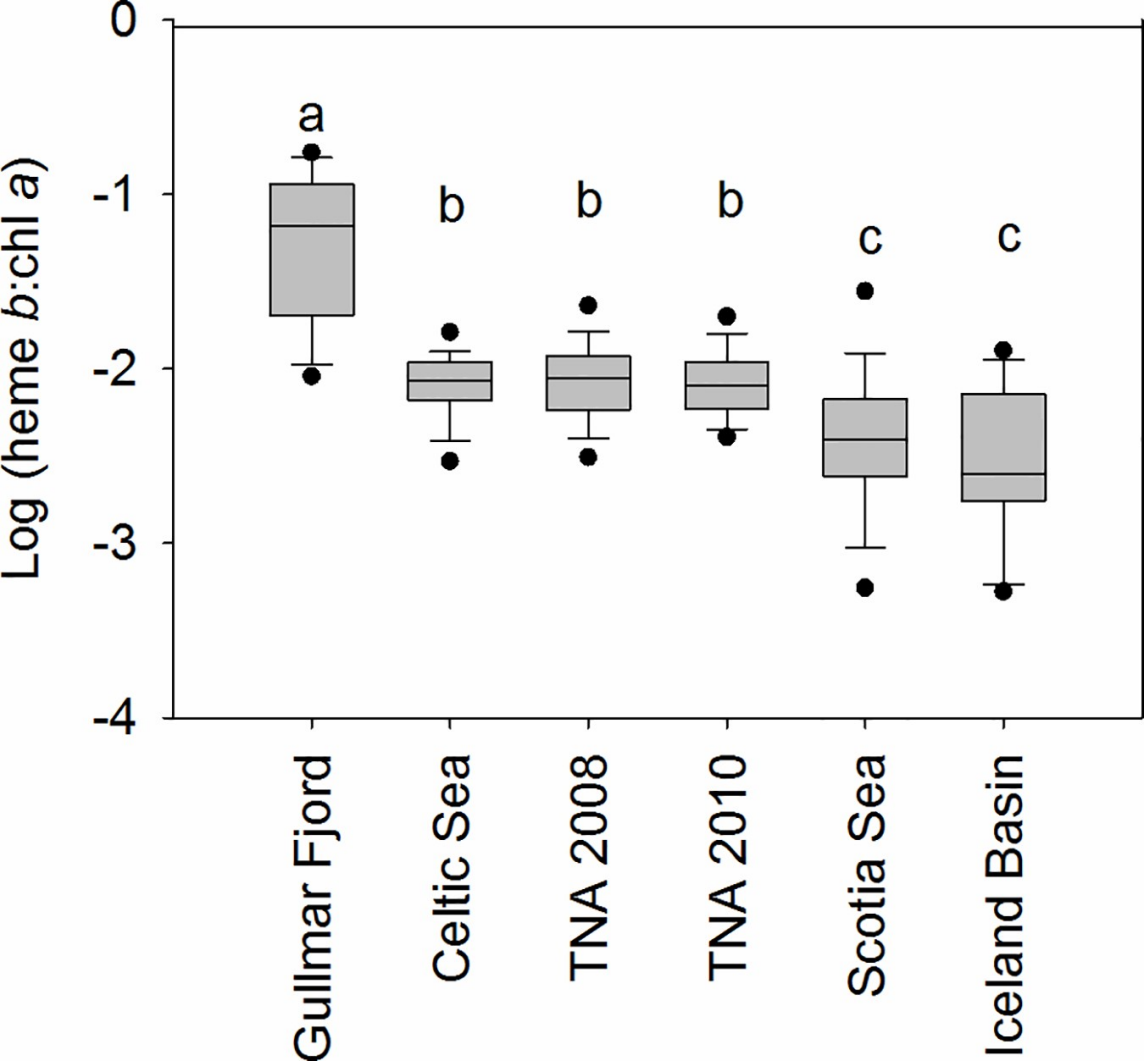
Box and whisker plot for log(heme *b*: chlorophyll *a*) values reported. From Gullmar Fjord (this study, n = 19), Celtic Sea (n = 27), (sub)-tropical North Atlantic in 2010 (n = 377; [6]) tropical North Atlantic in 2008; (n = 268), Scotia Sea (n = 34) and the Iceland Basin (n = 83, [12]). Black circles indicate the 5^th^/95^th^ percentile for log(heme *b*: chlorophyll *a*). Letters denote significantly different groups (one way non parametric ANOVA, p<0.01).

Heme *b* concentrations have now been reported from the Celtic Sea, two studies in the (sub)-tropical and North Atlantic Ocean, the Iceland Basin in the high latitude North Atlantic and in the Southern Ocean. Table 2 summarises heme *b* concentrations reported in previous studies, together with chl *a* and iron concentrations. The peak value of heme *b* obtained in the Gullmar Fjord itself in this study was 161 pmol L^-1^, which was approximately 8 times higher than the previous maximum, recorded downstream of St. Georgia in the Scotia Sea [12]. The Gullmar Fjord is the most iron replete environment in which heme *b* has been quantified and our results thus suggest a substantial increase in the iron containing hemoproteins determined using our technique is possible in marine environments typified by higher iron concentrations. Accordingly, fjord heme *b*: TPC ratios were comparable to iron replete phytoplankton cultures [6] and heme *b* was also higher relative to chl *a* than previously reported, especially after the decline in chl *a* post bloom. Fig 6 shows a box and whisker plot of the log(heme *b*: chl *a*) ratio for the data obtained in this study together with all previously reported field data. A non parametric ANOVA on ranks indicated that log(heme *b*: chl *a*) ratios were significantly higher in the Gullmar Fjord compared to all previously published data, while log(heme *b*: chl *a*) values were significantly lower in the Scotia Sea and Iceland Basin. The data reported in this study suggest that neither TPC nor chl *a* correlated with heme *b* in the fjord proper. Additionally, previous studies occurred in nutrient limited regimes [6, 12] so that changes observed between these studies are unlikely to be overly influenced by any changes in heme *b* occurring during initial growth of a primary bloom. Some variability between and within studies will be a result of the relative degree of nitrate or phosphate depletion and likely also a reflection of changes in community composition. However, our data suggest that the greatest influence on heme *b* abundance relative to bulk biomass properties such as chl *a* in the studies published to date, was the concentration of iron in the water column. The heme *b* pool (determined with the ammoniacal extraction method) therefore represents a relatively plastic iron pool, which can be reduced, via adaptation [13] or acclimation [6], when iron availability decreases. The reduction in this heme *b* protein pool reflects an increase in heme growth efficiency (HGE; [13]). Such an increase in HGE could be linked to an overall reduction in iron use [42] and/or a reallocation of resources away from heme *b* and towards other iron containing proteins as has been observed with nitrate reductase [43]. Whichever is the case, our finding that near shore coastal water communities contain higher heme *b* concentrations than observed even in iron replete open ocean environments [6, 12] confirms that the iron metabolism of microbial communities in the marine environment is consistently tuned to the ambient iron supply.

**Table 2:** Comparison of heme *b*, chlorophyll *a* and dissolved iron values observed in the Gullmar Fjord (this study) with other studies from the Atlantic Ocean.

Study area	Heme *b* (pmol L^-1^)	Chlorophyll *a* (nmol L^-1^)	Dissolved iron (nmol L^-1^)
Iceland Basin	1.1±0.7^[^^12^^]^	1.2±0.8^[^^12^^]^	<0.03–0.22^[^^44^^]^
Scotia Sea	5.1±4.8^[^^12^^]^	1.9±1.7^[^^12^^]^	<0.03–0.6^[^^45^^]^
Tropical North Atlantic	2.3±1.7^[^^12^^]^	0.25±0.13^[^^12^^]^	<0.1–0.37^[^^46^^]^
Celtic Sea	3.8±1.7^[^^6^^]^	0.5±0.3^[^^6^^]^	0.8–2.1^[^^47^^]^
Gullmar Fjord (this study)	66±45	1.2±0.6	4–40^[^^36^^,^ ^37^^]^

Heme *b* and chl *a* are expressed as averages ± standard deviation. Dissolved iron data are expressed as a range.

## 5. Conclusions

This study is the first to report heme *b* concentrations in an iron replete marine pelagic community over the progression of a naturally occurring spring bloom. Heme *b* concentrations were considerably greater in the mesocosms than previously reported elsewhere, likely a reflection of high levels of iron supply. The abundance of heme *b* relative to other bulk biomass properties such as chl *a* and POC changed over the course of the phytoplankton bloom, with heme *b* reaching a maximum earlier than both chl *a* and POC. Heme *b*: POC peaked at the onset of the primary bloom, but rapidly decreased again and remained relatively stable thereafter until the end of the experiment suggesting that heme *b* production may be elevated in the early, nutrient replete, stages of a bloom. As heme *b* is a significant component of the intracellular iron pool, this has potential implications for iron requirements and the intracellular allocation of iron during the early stages of phytoplankton blooms. The abundance of heme *b* relative to chl *a* was influenced by nutrient availability and changes in community composition resulting from reduced nitrate availability. This could be connected to the requirement for eukaryotic nitrate reductase, which contains heme *b*. This KOSMOS experiment resulted in no significant broad scale effects of high *p*CO_2_ (ca. 1000 μatm) upon heme *b* concentration relative to chl *a* or POC. However, the KOSMOS mesocosms are iron replete systems and this result does not exclude the potential for higher *p*CO_2_ to impact on iron containing biogenic compounds such as heme *b* in lower iron environments. Further studies on the impact of ocean acidification on heme *b* and iron requirements in low iron environments are therefore needed.

Heme *b* concentrations in Gullmar Fjord were also higher than those observed in previous studies as was the abundance of heme *b* relative to chl *a*. Comparison with previously published data suggests that the distribution of heme *b* relative to chl *a* is closely linked to the concentration of iron, so that there is a lower abundance of heme *b* relative to chl *a* in regions where iron is deplete (Fig 6). These findings thus have potential implications for the way in which phytoplankton utilise iron in the ocean, and the allocation of this important limiting nutrient towards protein pools driving different biogeochemical processes.

## Supporting information

S1 Table Chlorophyll *a*, nitrate+nitrite: Phosphate ratio, heme *b*, particulate organic carbon and particulate organic nitrogen values observed in samples collected during the course of the mesocosm experiment carried out in Gullmars fjord. Values represent the average ± the standard deviation of 10 individual mesocosms. N.D.–parameter was not determined on that day, <d.l.–less than the detection limit.
(DOCX)

S2 Table Chlorophyll *a*, nitrate+nitrite: Phosphate ratio, heme *b*, particulate organic carbon and particulate organic nitrogen values observed in samples collected in Gullmars fjord. N.D.–parameter was not determined on that day, <d.l.–less than the detection limit.
(DOCX)
