# Supplementary material for: Abundance of the iron containing biomolecule, heme b, during the progression of a spring phytoplankton bloom in a mesocosm experiment
Source: PLoS One. 2017 Apr 20;12(4):e0176268. doi: 10.1371/journal.pone.0176268 (PMC5398680; doi:10.1371/journal.pone.0176268)
Supplement: S1 Table — Values represent the average ± the standard deviation of 10 individual mesocosms. N.D.–parameter was not determined on that day, <d.l.–less than the detection limit. (DOCX) [file pone.0176268.s001.docx]

S1 Table. Chlorophyll *a*, nitrate+nitrite: phosphate ratio, heme *b*, particulate organic carbon and particulate organic nitrogen values observed in samples collected during the course of the mesocosm experiment carried out in Gullmars fjord. Values represent the average ± the standard deviation of 10 individual mesocosms. N.D. – parameter was not determined on that day, <d.l. – less than the detection limit.

| Date | Day after mesocosm closure | Chlorophyll *a* (nmol L^-1^) | Nitrate+nitrite:phoshate ratio | Heme *b* (pmol L^-1^) | Particulate organic carbon (µmol L^-1^) | Particulate organic nitrogen (µmol L^-1^) |
| --- | --- | --- | --- | --- | --- | --- |
|  |  |  |  |  |  |  |
| 10/03/2013 | 1 | 0.40±0.02 | 9.52±0.58 | N.D. | 13±2 | 1.9±0.3 |
| 12/03/2013 | 3 | 0.48±0.07 | 9.81±0.59 | N.D. | 17.3±4 | 2.3±0.5 |
| 14/03/2013 | 5 | 0.62±0.04 | 9.29±0.57 | N.D. | 12.9±2.4 | 1.8±0.3 |
| 16/03/2013 | 7 | 0.71±0.05 | 9.08±0.66 | N.D. | 14.7±1.7 | 1.8±0.3 |
| 18/03/2013 | 9 | 0.71±0.06 | 8.42±0.75 | 27±4 | 14.1±1.8 | 2.0±0.2 |
| 20/03/2013 | 11 | 0.68±0.07 | 8.89±0.68 | N.D. | 14.7±1.5 | 1.9±0.2 |
| 22/03/2013 | 13 | 0.67±0.07 | 8.90±0.23 | 55±14 | 17.5±2.4 | 2.2±0.4 |
| 24/03/2013 | 15 | 0.78±0.12 | 9.57±0.11 | N.D. | 16.4±1.3 | 2.3±0.2 |
| 26/03/2013 | 17 | 0.86±0.15 | 9.61±0.29 | 66±28 | 14.5±0.9 | 2.1±0.2 |
| 28/03/2013 | 19 | 1.03±0.22 | 9.49±0.24 | N.D. | 17.8±2.3 | 2.6±0.5 |
| 30/03/2013 | 21 | 1.26±0.35 | 9.15±0.29 | 92±39 | 17.1±1.4 | 2.7±0.3 |
| 01/04/2013 | 23 | 1.68±0.57 | 9.20±0.39 | N.D. | 18.6±1.4 | 3.3±0.3 |
| 03/04/2013 | 25 | 2.18±1.04 | 8.22±0.78 | 753±435 | 20.3±3.1 | 3.3±0.7 |
| 05/04/2013 | 27 | 3.10±1.20 | 6.58±1.86 | N.D. | 25.0±4.0 | 4.4±0.9 |
| 07/04/2013 | 29 | 4.38±1.18 | 3.72±1.89 | 894±929 | 32.2±5.1 | 5.7±0.9 |
| 09/04/2013 | 31 | 4.27±1.49 | 3.51±4.34 | N.D. | 42±9 | 6.4±1.9 |
| 11/04/2013 | 33 | 4.52±1.39 | 2.1±1.71 | 328±66 | 51±14 | 6.7±0.8 |
| 13/04/2013 | 35 | 3.79±1.47 | 1.65±1.39 | N.D. | 49±13 | 6.6±0.6 |
| 15/04/2013 | 37 | 2.92±1.11 | 1.07±0.44 | 113±43 | 51±14 | 7.0±0.6 |
| 17/04/2013 | 39 | 1.71±0.69 | 1.22±1.55 | N.D. | 40±13 | 5.6±0.7 |
| 19/04/2013 | 41 | 1.66±0.49 | 0.66±0.54 | 141±18 | 41±11 | 6.1±0.9 |
| 21/04/2013 | 43 | 2.02±0.48 | 0.39±0.30 | N.D. | 46±11 | 6.9±1.2 |
| 23/04/2013 | 45 | 2.08±0.57 | 0.33±0.17 | 100±33 | 48±11 | 6.9±1.3 |
| 25/04/2013 | 47 | 2.82±0.63 | 0.53±0.18 | N.D. | 40±10 | 5.2±1.0 |
| 27/04/2013 | 49 | 3.53±0.84 | 0.57±0.68 | 158±36 | 49±11 | 6.9±1.0 |
| 29/04/2013 | 51 | 3.72±0.84 | 2.22±5.19 | N.D. | 52±12 | 7.1±0.9 |
| 01/05/2013 | 53 | 3.66±1.26 | 2.29±2.50 | 171±34 | 52.7±13 | 7.1±1.2 |
| 03/05/2013 | 55 | 3.80±1.09 | 4.22±5.62 | N.D. | 45±12 | 5.0±0.8 |
| 05/05/2013 | 57 | 3.75±0.86 | 2.33±1.47 | 135±38 | 46±12 | 5.6±0.8 |
| 07/05/2013 | 59 | 3.15±0.85 | 1.33±1.12 | N.D. | 43±12 | 5.0±0.7 |
| 09/05/2013 | 61 | 2.49±0.83 | 1.46±0.74 | 144±21 | 42±12 | 5.5±0.9 |
| 11/05/2013 | 63 | 2.13±0.68 | 1.25±0.90 | N.D. | 40±13 | 5.1±1.1 |
| 13/05/2013 | 65 | 2.02±0.64 | 1.33±0.94 | 109±31 | 38±13 | 4.7±0.9 |
| 15/05/2013 | 67 | 1.86±0.58 | 1.35±1.15 | N.D. | 37±12 | 4.7±1.0 |
| 17/05/2013 | 69 | 1.58±0.55 | 0.93±0.53 | 64±37 | 35±11 | 4.2±0.7 |
| 19/05/2013 | 71 | 1.28±0.46 | 1.55±2.04 | N.D. | 31±9 | 4.0±0.5 |
| 21/05/2013 | 73 | 1.20±0.40 | 0.93±1.08 | 111±19 | 30±8 | 3.7±0.8 |
| 23/05/2013 | 75 | 0.89±0.27 | 0.69±0.63 | N.D. | 26±7 | 3.6±0.6 |
| 25/05/2013 | 77 | 0.84±0.29 | 0.89±0.68 | 115±25 | 25±7 | 3.1±0.5 |
| 27/05/2013 | 79 | 0.85±0.26 | 0.82±0.56 | N.D. | 27±8 | 3.0±0.5 |
| 29/05/2013 | 81 | 0.89±0.28 | 0.78±0.91 | 158±82 | 31±6 | 3.4±0.5 |
| 31/05/2013 | 83 | 0.76±0.29 | 0.40±0.32 | N.D. | 25±6 | 2.7±0.4 |
| 02/06/2013 | 85 | 0.65±0.35 | 0.49±0.21 | 89±56 | 23±5 | 2.6±0.3 |
| 04/06/2013 | 87 | 0.85±0.36 | 1.17±1.56 | N.D. | 25±8 | 2.6±0.3 |
| 06/06/2013 | 89 | 0.81±0.27 | 1.91±1.30 | 73±43 | 26±6 | 3.0±0.5 |
| 08/06/2013 | 91 | 0.70±0.22 | 1.58±0.95 | N.D. | 27±7 | 2.7±0.3 |
| 10/06/2013 | 93 | 0.67±0.16 | 1.41±1.08 | 119±65 | 29±6 | 3.1±0.6 |
| 12/06/2013 | 95 | 0.62±0.15 | 0.45±0.21 | N.D. | 25±5 | 2.9±0.5 |
| 14/06/2013 | 97 | 0.62±0.21 | N.D. | 174±62 | 26±7 | 3.1±0.6 |
