# Supplementary material for: Abundance of the iron containing biomolecule, heme b, during the progression of a spring phytoplankton bloom in a mesocosm experiment
Source: PLoS One. 2017 Apr 20;12(4):e0176268. doi: 10.1371/journal.pone.0176268 (PMC5398680; doi:10.1371/journal.pone.0176268)
Supplement: S2 Table — N.D.–parameter was not determined on that day, <d.l.–less than the detection limit. (DOCX) [file pone.0176268.s002.docx]

S2 Table. Chlorophyll *a*, nitrate+nitrite: phosphate ratio, heme *b*, particulate organic carbon and particulate organic nitrogen values observed in samples collected in Gullmars fjord. N.D. – parameter was not determined on that day, <d.l. – less than the detection limit.

| Date | Day after mesocosm closure | Chlorophyll *a* (nmol L^-1^) | Nitrate+nitrite: phoshate ratio | Heme *b* (pmol L^-1^) | Particulate organic carbon (µmol L^-1^) | Particulate organic nitrogen (µmol L^-1^) |
| --- | --- | --- | --- | --- | --- | --- |
|  |  |  |  |  |  |  |
| 10/03/2013 | 1 | 1.44 | 10.32 | N.D. | 19.1 | 2.9 |
| 12/03/2013 | 3 | 1.84 | 10.94 | N.D. | 23.9 | 3.6 |
| 14/03/2013 | 5 | 1.65 | 9.89 | N.D. | 17.3 | 2.4 |
| 16/03/2013 | 7 | 1.94 | 10.66 | N.D. | 58.3 | 5.7 |
| 18/03/2013 | 9 | 0.39 | 5.55 | 20.61 | 42.3 | 3.8 |
| 20/03/2013 | 11 | 2.28 | 4.23 | N.D. | 32.7 | 4.2 |
| 22/03/2013 | 13 | 1.05 | 8.74 | 15.92 | 26.6 | 3.2 |
| 24/03/2013 | 15 | 1.51 | 2.74 | N.D. | 34.0 | 4.0 |
| 26/03/2013 | 17 | 1.14 | 8.19 | 26.33 | 27.4 | 3.3 |
| 28/03/2013 | 19 | 1.59 | 5.72 | N.D. | 37.9 | 5.1 |
| 30/03/2013 | 21 | 1.00 | 7.72 | 46.24 | 25.4 | 3.3 |
| 01/04/2013 | 23 | 0.93 | 3.18 | N.D. | 24.9 | 3.2 |
| 03/04/2013 | 25 | 1.01 | 7.75 | 59.17 | 21.9 | 2.4 |
| 05/04/2013 | 27 | 3.94 | 6.61 | N.D. | N.D.32.9 | 4.2 |
| 07/04/2013 | 29 | 2.14 | 6.13 | 50.42 | N.D.25.3 | 3.7 |
| 09/04/2013 | 31 | 2.67 | 3.74 | N.D. | N.D.24.7 | 3.4 |
| 11/04/2013 | 33 | 0.77 | 2.44 | 32.25 | 17.7 | 2.4 |
| 13/04/2013 | 35 | 0.71 | 14.9 | N.D. | 18.5 | 2.4 |
| 15/04/2013 | 37 | 0.59 | 1.18 | 59.12 | 19.0 | 3.0 |
| 17/04/2013 | 39 | 0.36 | 1.0 | N.D. | 22.0 | 3.8 |
| 19/04/2013 | 41 | N.D. | 10.25 | 42.66 | 22.0 | 3.1 |
| 21/04/2013 | 43 | 0.37 | 7.55 | N.D. | 27.1 | 4.4 |
| 23/04/2013 | 45 | 0.49 | 6.31 | 51.11 | 27.0 | 4.0 |
| 25/04/2013 | 47 | 0.48 | 6.82 | N.D. | 27.1 | 4.8 |
| 27/04/2013 | 49 | 0.65 | 3.80 | 54.36 | 25.2 | 3.6 |
| 29/04/2013 | 51 | 1.22 | 12.2 | N.D. | 338 | 5.2 |
| 01/05/2013 | 53 | 1.27 | 9.06 | 143.79 | 27.0 | 4.9 |
| 03/05/2013 | 55 | 1.31 | 39.2 | N.D. | 29.5 | 4.5 |
| 05/05/2013 | 57 | 1.15 | 0.54 | 73.19 | 25.6 | 4.5 |
| 07/05/2013 | 59 | 0.98 | 19.9 | N.D. | 25.4 | 4.5 |
| 09/05/2013 | 61 | 1.23 | 14.5 | <d.l. | 26.8 | 4.4 |
| 11/05/2013 | 63 | 1.22 | 6.7 | N.D. | 28.4 | 5.4 |
| 13/05/2013 | 65 | 0.91 | 6.6 | <d.l. | 18.3 | 3.5 |
| 15/05/2013 | 67 | 1.21 | 10.8 | N.D. | 24.5 | 4.4 |
| 17/05/2013 | 69 | 0.98 | 7.9 | <d.l. | 22.5 | 3.5 |
| 19/05/2013 | 71 | 0.95 | 9.8 | N.D. | 28.0 | 4.2 |
| 21/05/2013 | 73 | 1.11 | 2.9 | 131.80 | 22.1 | 2.9 |
| 23/05/2013 | 75 | 0.76 | 1.0 | N.D. | 22.1 | 3.3 |
| 25/05/2013 | 77 | 0.73 | 3.0 | 100.90 | 16.5 | 2.5 |
| 27/05/2013 | 79 | 0.62 | 3.8 | N.D. | 21.3 | 3.3 |
| 29/05/2013 | 81 | 0.92 | 2.8 | 49.23 | 21.7 | 2.8 |
| 31/05/2013 | 83 | 0.74 | 1.9 | N.D. | 25.6 | 3.4 |
| 02/06/2013 | 85 | 0.82 | 2.1 | 161.64 | 19.6 | 2.4 |
| 04/06/2013 | 87 | 1.41 | 1.8 | N.D. | 25.9 | 3.9 |
| 06/06/2013 | 89 | 1.45 | 2.3 | 19.46 | 22.3 | 3.3 |
| 08/06/2013 | 91 | 0.96 | 1.3 | N.D. | 27.8 | 3.6 |
| 10/06/2013 | 93 | 0.71 | 1.8 | 152.69 | 26.1 | 3.6 |
| 12/06/2013 | 95 | 1.43 | 1.7 | N.D. | 29.9 | 4.2 |
| 14/06/2013 | 97 | 1.25 | N.D. | 48.40 | 24.9 | 3.5 |
